# A Memoir on Those Diseases Which Proved so Fatal to Our Troops during the Burman War; with a Comparative Sketch of Their Analogous Cases in Hindostan, during a Service of Some Years

**Published:** 1829-03

**Authors:** James Walsh

**Affiliations:** Assistant Surgeon 89th Regiment.


### DISEASES IN HINDOSTAN.

*A Memoir on those Diseases which proved so fatal to our Troops during the Burman War; with a comparative Sketch of their analogous Cases in Hindostan, during a Service of some years.* By James Walsh, Assistant Surgeon 89th Regiment.

(Concluded from p. 115.)

Such was the general character of disease prevading nearly the whole of our force in the Pegu Delta and province of Lower Siam for the first few months; but, towards the termination of the monsoon, affairs put on an improving appearance. A more gratifying order of things now took place, a better and more varied supply of all the necessaries of life began to meet the eye, and to cheer our hitherto desponding minds.

The weather, too, becoming dry, pleasant, and cool, so powerfully contributed to the improvement of our general health and feelings, as enabled the force to undergo, with little comparative illness, the incessant military operations from November 1824 to the following January, which were called for by the imposing attitude and immense force of the Burmans, then collected about Rangoon, as they flattered themselves, for our total extermination.

Those barbarian hordes being, however, broken down and dispersed, we at length (February 1825) commenced the long-projected advance towards the interior.

Our progress was necessarily slow, as the two divisions of our force, one by land, and the other in boats, had to communicate occasionally, thus giving rise to much delay. The obstacles, also, to the advance of the water column were sometimes so great and formidable as to be productive of casualties and illness to no small extent.

The illness, being the effect of the new circumstances in which the troops were placed, had a somewhat diversified appearance.

Cooped up in boats for weeks, without bedding, or adequate protection from the heavy, raw, and chilling dews of the night; exposed, on the other hand, during the day to a powerful sun, with a very insufficient awning; and, to wind up the climax of uncomfortable accommodation, they were generally so crowded that the whole space could scarcely afford more room than two and a half feet by one to each man!

Thus much for the boats more immediately military; while the subordinate staff, commissaries, and their clerks, pilot lads, or lead boys, as they are called, now commanding boats, or divisions of boats, might be seen in large and decked vessels, with commodious cabins, or other comfortable and ample accommodation; yet at the same time a boat could not be obtained for the sick or wounded without some difficulty and delay.

The soldiers were attended, and supplied with medicine, in the boats wherein they were originally crowded, as often as I could procure a canoe, or other means of visiting them. On one occasion, a case of cholera was reported from a distant boat; but, although every effort was made on my part, no conveyance could be obtained for hours, when it was too late; as I found the man in the last stage of collapse, and dissolution so fast approaching that he was nearly incapable of taking medicine.

Officers, also, some of whom laboured under severe and complicated disease, could not have from me that degree of attention their cases required, and which I was anxious to pay, in consequence of my not having been furnished with a boat for this indispensable purpose, notwithstanding my urgent and repeated applications to that effect. At length, when the voyage had considerably advanced, a vessel was fitted up for hospital purposes; and, although its means of accommodation were far too limited, it was still of advantage, as it was capable of receiving a few of the very worst cases, and so far of lessening the already overcrowded state of the other boats.

We were by this time drawing towards Prome, and all hearts were elated with the hope of again encountering the enemy; and, under the improved diet, and continuance of favorable weather, or N.E. monsoon, a corresponding change in the nature of morbid action began to show itself extensively, in spite of the untoward circumstances lately noticed. Disease became less frequent, and, although cholera and remittent fever would sometimes show themselves, they were in general of a less fatal description than what took place in the low country.

On the return, however, of the wet monsoon, which set in soon after the capture of Prome by our troops, in April 1825, sickness manifested itself with renewed violence and frequency, but by no means with the extensive mortality experienced in lower Pegu, although several deaths took place.

During this second rainy season the troops were very little called upon for military exertion; of course, illness and a fatal result were proportionably lessened. The country, also, about this time yielded an abundant supply of fresh provisions, beef, poultry, and vegetables; the immediate effect of that freer intercourse with the natives, which we were at length able to establish. The general state of the force, with respect to comfort and equipment, became, therefore, so much improved, that the troops looked forward with perfect confidence to the result of the expected renewal of hostilities, notwithstanding their amazing numerical inferiority to the Burmans crowding about them.

Military operations were recommenced by the European part of the force on the 1st December, 1825, and continued with triumphant success up to the 6th, when an enemy could no longer be found till our arrival at Patangow early in the following January, after a forced and harassing march of about 250 or 300 miles. In these first days of success, a great degree of exposure, hardship, and privation was necessarily undergone, and yet was not succeeded by illness in any shape, at least for some days.

It was deemed advisable not to allow the tents or baggage to accompany the force during the continuance of these operations; consequently, every one had to bivouac as well as he could, some on very high ground, surrounded by swamps, and with an unusual degree of cold at night; others on ground rather low, and as yet not perfectly dry; all exposed to nocturnal fogs, chilling, dense, and thick enough to give a thorough wetting; the thermometer at this time ranging from fifty to sixty degrees at night, to upwards of one hundred in the middle of the day. Yet all this was borne, I believe, with a general exemption from illness for the time; which must, no doubt, have been the effect of the previous excitation and triumph so generally felt.

But this state of things at length subsided: excitement, no longer kept up by the presence of the enemy, soon sank into exhaustion and debility; to which intoxication, and irregularity in the distribution of rations, must have materially contributed; thus affording an ample field for the influence of a powerful morbific agency, necessarily arising from extreme and rapid atmospheric vicissitude, combined with strong miasmal impregnation.

After our first movement in the pursuit, a halt for two or three days was necessary, to bring up our baggage and *matériel* from Prome. Ground for an encampment was therefore selected, and, with our usual infelicity, the tents were pitched in a low flat, with a surface of slimy mud, by no means fully dried, or freed from rice straw in a state of decomposition. The powerful action of a few days' sun had formed large and numerous fissures in this ground, presenting to the medical eye so many channels for poisonous exhalation. To complete the picture, it rained so heavily for some time, that the surface became soft and miry throughout, and in some places covered with water.

Here cholera of the old character again renewed its ravages, surpassing, if possible, in the fatal rapidity of its collapse, the former visitation in the Pegu Delta. Sixteen, twenty, or more, were, perhaps, sent into Prome, about twelve miles distant, in one day; as the purposed rapidity of the pursuit admitted of as little incumbrance as possible. Some died on the way; others while being put on the miserable dhoolies, or conveyances for their transport; and all, or the much greater part, I believe, sooner or later.

Another halting place was differently circumstanced, but by no means less productive of sickness, although of another kind. The tent flags, and of course the tents, were placed on the slopes of some very high rising ground, but so near their base that several were necessarily in the soft ooze formed by the surface water or springs descending from these hills to an extensive swamp, within forty or fifty yards of the camp, and exhibiting most abundantly all the materials for the extrication of a highly concentrated and virulent miasm. A different morbid action now evinced itself. Cholera had by this time nearly subsided, or had rather, perhaps, exhausted itself by the destruction of its victims, and was succeeded by remittent fever to such an extent, that upwards of 150 were to be carried in every possible way, on gun carriages, carts, boats when near the river, &c. As for dhoolies, the usual conveyance for sick on a march in India, where they are liberally furnished and equipped, at least in the Madras presidency, the number allotted on this occasion to the 89th may have been ten or twelve! and several of these were so broken down as often to give way, and tumble out the unfortunate soldier, already sufficiently suffering under complicated injury or disease. In this way, too, the soldier might be observed to sink in death, exhausted by a repetition of the tumbling, as well as by exposure to the sun, owing to the imperfect construction of machines, which in India have all the advantage and comfort of the more costly palankeen.

At length the force reached Patanagow, on the east bank of the Irriwaddy, directly opposite to, and in a great measure commanding, the extensive fortifications of Mallown.

A treaty was entered upon immediately; and, during the time which elapsed in its discussion and expected ratification, fever manifested itself to as great an extent as before; in many cases with excessive violence, and in all with much obstinacy, as well as with great irregularity and variety; but universally of the remittent or intermittent form, evidently arising from jungle or marsh miasm. The violent remittent appeared to be the effect of jungle effluvia or impregnation; while those more immediately in the neighbourhood of the swamp or marsh would generally put on the intermittent type.

About half of the officers (seven or eight), with upwards of two hundred men, out of three hundred with whom our march was commenced, were at this time attacked with no small violence. Two of the officers soon sank, while the progress of the others towards recovery was extremely slow, and attended with much difficulty, as well as with a disposition to frequent relapse. The mortality among the men approached nearly to that which had taken place lower down; and, altogether, such was the result of illness, casualty, and death, that the battalion, when setting out for Pagham-mew, after the capture of Mallown, could only muster three or four subaltern officers, and about eighty men for duty! out of nearly one thousand who originally came over, or afterwards joined.

The treatment of these jungle fevers required very little deviation from what had hitherto been practised in the alluvial remittent of the low country; but, as the sensorial disturbance and general excitement in many cases appeared likely to lead more rapidly to disorganization, temporal arteriotomy was more frequently employed, and that even as a substitute for leeches, now no longer to be procured. It was observed to be attended with a more rapid diminution of high vascular reaction than venesection in general produced; the violence also of cephalic determination was seen to yield more satisfactorily when it could be fully and promptly performed. But, although not always procuring the wished-for extent of depletion, owing to bad lancets or other unavoidable circumstances, it always evinced effects superior to those of any other mode of topical blood-letting that could be attempted. When free depletion and purgatives had sufficiently lowered and prepared the system, mercurial action was promoted as quickly as possible, and with the best effects. Soon after subjecting the patient to this active treatment, the febrile attack would occasionally intermit as before, or nearly subside, when the cinchona was given with success. But so great had been the consumption, and so general the demand for this remedy, that an adequate supply was not always to be obtained. In this unfortunate predicament, the exhibition of the medicine was necessarily limited, and too often the progressive convalescence, previously so promising, would be retarded, or perhaps wholly lost.

As the facilities of communication and conveyance by water had by this time much improved, and as the troops were preparing for a further (and their last) advance, the more grave and serious cases were selected and sent down to Prome; the less serious and the convalescent were brought up the river, with the portion of the force proceeding by that route. During this change of conveyance and removal from the scene of the original morbid impregnation, the conclusions previously laid down were more strongly confirmed; as the generality of the cases so forwarded soon assumed a marked change of type, with every promise of increasing amelioration; except in those who had previously laboured under great and obstinate functional derangement, or incipient disorganization of some viscus, for whom, alas! no hope could be anticipated from any possible change of scene, circumstances, or treatment.

The contest had by this time been drawing towards a close, and the progress of our little army up the country at length ceased, on the enemy having finally acceded to those propositions, into the discussion of which they had entered on two occasions before, but with much more of finesse and subterfuge than with any purpose of acquiescence, till impressively convinced of its urgent necessity by their repeated and signal defeats, and by our near approach to their capital.

Of the still more frightful mortality of Aracan, I had no direct or satisfactory conception, as my range of service in the Burman empire was confined to its provinces of Pegu, Lower Siam, and Ava. Appalling, and almost incredible as our losses and privations were in the first and latter of these countries, I have had reason to infer that they were, if possible, surpassed in that portion of our army serving in Aracan; although, at the commencement, the troops there were said to have been better supplied, and in a great measure exempt, at least for a time, from the visitation by which we were so rapidly and fatally assailed.

At Tavoy and Mergui, the principal places of Lower Siam to which my regiment was despatched for some weeks, before our first movement towards the Burman capital, we experienced an unexpected and fortunate change of circumstances, so different from what we underwent a short time previously at Rangoon, that the most beneficial consequences ensued. Disease, which had made such ravages up to this period, now began to confine itself to those chronic or relapsed cases, principally of scorbutic dysentery and intermittent fever, which had been allowed to accompany the expedition, chiefly with the hope of deriving benefit from the voyage and change of scene. This expectation was in some measure realized, as some of the hitherto hopeless cases soon began to put on an improving appearance, and a greater disposition to yield to medicine and regimen.

Tavoy, which surrendered on our approach, afforded supplies of almost every thing that could be wished for, an abundance of poultry, fish, fresh beef, and pork, with a great variety of tropical fruits and vegetables; to nearly all of which we had for months been strangers. A good understanding, too, was soon established with the natives, a mixed race of the old Siamese with their Burman conquerors, who freely gave up the produce of their farms for our subsistence, and often for an inadequate compensation. The climate, although within thirteen degrees of the equator, was found infinitely superior to that of Rangoon, Calcutta, or Madras, and might be said to resemble, or even to excel, the general temperature of the Malabar coast. Although so much more to the southward, the heat was by no means so great as in those last-mentioned places, the thermometer at this time of the year ranging gradually from seventy to eighty-six; nor could atmospheric vicissitude be observed at any time to a sudden or considerable extent. The general aspect of the country was rather wild and uncultivated, but still many places might be found beautifully romantic and diversified.

This may serve as a view of part of the interior, yet the approach to the town by water, or what is called its anchorage, (a space of about thirty miles,) was very different. The town and its neighbourhood proved delightful and healthy, but the anchorage seemed much the reverse. Surrounded by an amphitheatre of high peaked hills, terminating suddenly in abrupt or swampy valleys, all thickly covered over with wood and jungle, and having a few roods only of level or miry ground between the river and those hills towering one behind another, yet running more or less parallel to it, the exhalations were necessarily miasmal in a high degree; and so loaded was the atmosphere with a heavy dense vapour during a great part of the morning, that it required some hours of strong sun to clear it.

As the channel did not admit the passage of the larger ships to the town, the sick were all ordered on board the transport under my charge; yet, although a delay of three weeks took place in this unpromising situation, the deaths were comparatively few, and the admissions also were, in general, of the old and often relapsing cases.

As arrangements were in train for the permanent occupation of this and other districts, the troops were deriving the fullest benefit from the ample comfort and gratifying variety of accommodation so unexpectedly found there; and, with renovated health, and high spirits, proceeded to Mergui, the next point of attack; by no means so easy or bloodless a conquest.

This town is about one degree nearer the line, and proved still superior to Tavoy, presenting as pleasing and diversified a landscape as can well be imagined. Although nearly open to the sea, it is amply protected from its violence by an archipelago of islands, extending some way along the coast, and forming capacious channels to the immediate anchorage of Mergui, situated at the mouth of the Tanasserim river. This anchorage, as well as the town, additionally protected by other islands in its close neighbourhood, furnishes a highly picturesque and romantic view, admirably fitting it for the convalescent establishment formed there soon after its capture. Its position is so elevated, and generally so well cleared, as scarcely to admit of miasmal formation; and its having yielded an abundant and permanent supply of every necessary of life, adapted it more particularly for the reception of those cases of chronic illness or casualty, which did not seem to call for being immediately invalided, but which might still require a change of scene, if only as an advantageous removal from the more immediate neighbourhood of military operation.

